# Sum signal dosimetry: A new approach for high dose quality assurance with Gafchromic EBT3

**DOI:** 10.1002/acm2.12045

**Published:** 2017-02-02

**Authors:** Davide Cusumano, Maria Luisa Fumagalli, Francesco Ghielmetti, Linda Rossi, Giuliano Grossi, Raffaella Lanzarotti, Laura Fariselli, Elena De Martin

**Affiliations:** ^1^ School of Medical Physics University of Milan 20133 Milan Italy; ^2^ Health Department Fondazione IRCCS Istituto Neurologico Carlo Besta 20133 Milan Italy; ^3^ Department of Computer Science University of Milan 20135 Milan Italy; ^4^ Department of Neurosurgery Radiotherapy Unit Fondazione IRCCS Istituto Neurologico Carlo Besta 20133 Milan Italy

**Keywords:** dosimetric verification, Gafchromic EBT3, radiosurgery/hypofractioned radiotherapy

## Abstract

Gafchromic EBT3 film dosimetry in radiosurgery (RS) and hypofractionated radiotherapy (HRT) is complicated by the limited film accuracy at high fractional doses. The aim of this study is to develop and evaluate sum signal (SS) film dosimetry to increase dose resolution at high fractional doses, thus allowing for use of EBT3 for dose distribution verification of RS/HRT treatments. To characterize EBT3 dose–response, a calibration was performed in the dose range 0.44–26.43 Gy. Red (RC) and green (GC) channel net optical densities were linearly added to produce the SS. Dose resolution and overall accuracy of the dosimetric protocol were estimated and compared for SS,RC, and GC. A homemade Matlab software was developed to compare, in terms of gamma analysis, dose distributions delivered by a Cyberknife on EBT3 films to dose distributions calculated by the treatment planning system. The new SS and conventional single channel (SC) methods were compared, using 3%/1 and 4%/1 mm acceptance criteria, for 20 patient plans. Our analysis shows that the SS dose–response curve is characterized by a steeper trend in comparison with SC, with SS providing a higher dose resolution in the whole dose range investigated. Gamma analysis confirms that the percentage of points satisfying the agreement criteria is significantly higher for SS compared to SC: 95.03% vs. 88.41% (*P* = 0.014) for 3%/1 mm acceptance criteria and 97.24% vs. 93.58% (*P* = 0.048) for 4%/1 mm acceptance criteria. This study demonstrates that the SS approach is a new and effective method to improve dosimetric accuracy in the framework of the RS‐HRT patient‐specific quality assurance protocol.

## 1 Introduction

The recent technological evolution in radiation therapy has led to the development of new techniques in the treatment of neoplastic lesions utilizing high doses with extremely steep dose gradients and sub‐millimeter spatial accuracy. Although such progress has led to the reduction in the dose administered to healthy tissue, the clinical outcome relies heavily on the accordance between the dose calculated by the planning system and the dose actually delivered by the linear accelerator. The complexity of the new techniques thus makes it all the more necessary to evaluate such an accordance in doses.

Radiosurgery (RS) and hypofractionated radiotherapy (HRT) methods employ accurate imaging devices and dynamic delivery techniques to administer tightly conformed dose distributions while monitoring interfraction and intrafraction target positioning during the whole delivery process.

Gafchromic EBT3 films, thanks to their high spatial resolution, their near tissue equivalence, and their weak or absent energy and dose‐rate dependence, nowadays represent a widespread tool to assess complex dose distributions in high‐precision conformal radiotherapy where fractional doses of ~2 Gy are delivered.^1^, ^2^, ^3^ The use of Gafchromic EBT3 films in RS and HRT treatments (typical dose/fraction 5–21 Gy) is still under evaluation, as the EBT3 film response to high doses is characterized by a limited dose resolution. The characterization of the physical properties of EBT3 films has been described in detail in many studies for absorbed doses up to 40 Gy.^2^, ^4^ However, to our knowledge, the literature lacks information regarding patient dose distribution verification for doses higher than the red channel (RC) working range (~2–3 Gy).

Gafchromic EBT3 dosimetry is typically accomplished by the single channel (SC) method, which consists of using the RC data for doses below 10 Gy, and the green channel (GC) data for higher doses.^2^ However, sensitivity of the SC dose–response curve is still limited, with RC and GC signal values frequently associated with overlapping error bars when considering doses greater than 4 Gy. This reduces the accuracy of the resulting dosimetric analyses and the reliability of patient‐specific treatment plan verifications, and it is an obstacle to the use of Gafchromic EBT3 films for RS/HRT.

In 2011, Micke et al. proposed the triple‐channel method, a novel approach that uses all three color channels for uniformity deviation corrections, saving time, and avoiding switching of the color channel depending on the dose level.^5^, ^6^ Despite the several benefits offered by the triple‐channel method, studies by van Hoof et al. demonstrated that it produces the same level of accuracy as the RC with pre‐irradiation film scan.^7^

The aim of this study is to propose and validate a new and comprehensive dosimetric approach, by implementing the sum signal (SS) method in order to increase the film sensitivity at high doses, thus allowing the use of EBT3 for RS and HRT patient‐specific quality assurance (QA).

## 2 Methods

### 2.A Image processing

In order to use Gafchromic EBT3 films for absolute dosimetry, a preliminary dose calibration step was performed by irradiating 2.8 × 2.8 cm^2^ film pieces (batch #AO40411301) with a 6 MV photon beam**.**

Films were arranged in a solid water slab phantom, 5 cm deep from the phantom surface with a 15 cm solid water layer placed to produce backscattered radiation, and exposed perpendicularly to the Cyberknife beam axis (isocentric setup, source to axis distance = 80 cm, collimator diameter = 6 cm). An absolute dose measurement during irradiation was contextually performed according to IAEA TRS 398 protocol.^8^ An ionization chamber (Farmer FC65‐P, Scanditronix Medical AB, Uppsala, Sweden) was located 7 cm deep from the phantom surface and the chamber reading was then scaled to the film depth by applying a previously measured conversion factor. Two different films were simultaneously irradiated for each dose value in a range of 0.44–26.43 Gy.

The calibration films were digitized with the commercial flatbed scanner EPSON Expression 10000XL (Seiko Epson Corp, Nagano, Japan) before and 1 day after irradiation, with an image resolution of 150 dots per inch according to published recommendations.^4^, ^9^, ^10^ The scanner was always turned on at least 30 minutes before use and five preliminary scans without film on the scanner bed were performed in order to minimize the impacts of scanner noise and warm‐up effects of the scanner lamp.^9^, ^11^

Scans of the unirradiated and irradiated films were performed by positioning the film in the most uniform scanner region and acquiring the whole plate area in order to minimize the signal dispersion.^12^

Digitized images were analyzed using the ImageJ software (v1.39, National Institutes of Health, Bethesda, MD, USA) to obtain the mean pixel values before (PV_unexp_) and after irradiation (PV_exp_) in a 1 × 1 cm^2^ central region of interest (ROI). The obtained results were used to calculate the net optical density (netOD):

(1) $$netOD=OD_{\exp}-OD_{un\exp}=Log\left(\frac{PV_{un\exp}}{PV_{\exp}}\right)$$

In our new SS approach, the signal value to be associated with the corresponding calibration dose is given by the linear combination of the netOD values obtained for the red and green channels:

(2) $$SS=netOD_{RC}+netOD_{GC}$$

Blue channel is not included in equation (2) because its variation in optical density is not dependent on the absorbed dose, a fact which has been widely demonstrated in the literature and confirmed in our preliminary studies.^2^, ^5^ The calibration and analysis procedures was repeated with EBT3 films belonging to a new batch (#AO4041203) in order to study the reproducibility of the proposed method.

### 2.B Dose resolution analysis

The calibration data obtained in this study was used to quantitatively compare the new SS method with the red and green channel method in terms of the dose resolution achievable for dosimetry studies.

Several studies have linked the concept of dose resolution to the derivative of the calibration curve.^1^, ^13^, ^14^ This method produces only a qualitative estimation of the accuracy of the dose measurements. In order to obtain a quantitative estimation, the concept of dose resolution D_∆_^p^ as the minimal difference between two absorbed doses that allows them to be distinguished with a specified level of confidence *p*, developed by Baldock et al. for polymer gel dosimeters, was employed in this paper for Gafchromic dosimeters.^15^

In the specific mathematical formalism of this study, the dose values will be hereafter considered as the dependent variable (y_i_) and the signal values (as defined in eq. (1) for RC and GC; and in eq. (2) for SS) as the independent variable (x_i_).

Considering two consecutive calibration dose values y1 and y2, which differ by the quantity Δ= |y1‐y2|, the minimal detectable dose D∆p is defined as:

(3) $$D_{\Delta }^{p}=k_{p}\sqrt{u_{c}^{2}\left(y_{1}\right)+u_{c}^{2}\left(y_{2}\right)}$$

where k_p_ is a coverage factor equal to 1.96 for a 95% level of confidence and u_c_(y_i_) is the combined standard uncertainty of the dose values, which in local approximation is simply given by^15^:

(4) $$u_{c}^{2}\left(y_{i}\right)=\left[\frac{\partial y}{\partial x}\right]\sigma^{2}\left(x_{i}\right)$$

The uncertainties *σ*(x_,i,_) on signal values are examined in depth in section D for both the SC and SS methods. The applicability of the concept of local approximation and accurate estimation of the minimal detectable dose in this paper is ensured by the tight sampling during the calibration step of the dose range analyzed (0.44–26.43 Gy, 32 calibration points).

### 2.C Dose calibration

In order to calibrate the film response to dose the choice of the functional form better able to ensure a high accuracy level in the whole dose range investigated is of the utmost importance. Considering the results of published works, five functional forms were compared in this section: one rational function,^5^, ^6^ one double exponential function,^16^ and three polynomials.^16^, ^17^

The comparison was based on the use of the Akaike Information Criterion (AIC), a statistical method which allows to compare different non‐nested models on the basis of the best balance between accuracy requirements and number of fit parameters used.^18^ According to this criterion, the best fit function is the one showing the lowest AICc value*:*

(5) $$AICc=2k+\left[\sum_{i=1}^{n}\frac{{\left(y_{i,sper}-y_{i,fit}\right)}^{2}}{\left(n-k\right)\cdot \sigma_{eff}^{2}\left(y_{i}\right)}\right]+\frac{2k(k+1)}{n-k-1}$$

where k is the number of parameters in the fit function, y_i,sper_ is the dose value measured during the film calibration step, y_i,fit_ is the corresponding dose value obtained by the fit function investigated, and *σ*_eff_(y_i_) is the effective uncertainty:

(6) $$\sigma_{eff}^{2}\left(y_{i}\right)=\sigma^{2}\left(y_{i}\right)+{\left(\frac{\partial y}{\partial x}\right)}^{2}\sigma^{2}\left(x_{i}\right)$$

The effective uncertainty *σ*_eff_(y_i_) takes into account the experimental uncertainties *σ*(y_i_) associated with the dose values (equal to 1% of the measured value, according to the ionization chamber certificate) and the uncertainties *σ*(x_i_) associated to signal values.

For all functional forms investigated, the fit parameters were calculated using the effective variance method,^19^ since that the experimental uncertainties *σ*(x_i_) and *σ*(y_i_) were similar in size.

### 2.D Accuracy evaluation of the dosimetric protocol

The overall accuracy of the dosimetric protocol developed was investigated for both the SC and SS methods taking into account the various sources for error.^11^ In general, the uncertainties associated with the dose verification through the use of radiochromic films can be characterized into three main category sources^16^, ^17^:

1. uncertainties related to the signal value determination (Δ_meas_);
2. uncertainties related to intrafilm and interfilm uniformity (Δ_film_); and
3. uncertainties related to the fit procedure (Δ_fit_);

The size of uncertainties for the three sources listed above was assessed and statistically included in this study to obtain and investigate their overall accuracy associated with the dosimetric procedure:

(7) $$\Delta_{total}=\sqrt{\Delta_{meas}^{2}+\Delta_{film}^{2}+\Delta_{fit}^{2}}$$

The first type of uncertainty (Δ_meas_) includes errors due to the scanning procedure (warm‐up effects of the scanner lamp, uniformity and reproducibility in the scanner acquisition) and image analysis procedure (determination of net optical density starting from the pixel values).

In terms of mean percentage error, Δ_meas_ is equal to:

(8) $$\Delta_{meas}=\frac{100\%}{n}\sum_{i=1}^{n}\frac{\sigma (x_{i})}{x_{i}}$$

For the SC approach, *σ*(x_i_) is the uncertainty associated with the mean netOD value, which is obtained by averaging the netOD values of the two films exposed to the same dose:

(9) $$\sigma_{SC}\left(x_{i}\right)=\sqrt{\left(\frac{1}{2}\right)\delta {\left(netOD_{1}\right)}^{2}+{\left(\frac{1}{2}\right)}^{2}\sigma {\left(netOD_{2}\right)}^{2}}$$

Each calibration film is affected by an indetermination *δ*netOD_i_ obtained by applying the error propagation law to Eq. (1), 20, 21:

(10) $$\delta netOD=\sqrt{{\left(\frac{1}{ln10\cdot PV_{un\exp}}\right)}^{2}{\left(\delta PV_{un\exp}\right)}^{2}+{\left(-\frac{1}{ln10\cdot PV_{exp}}\right)}^{2}{\left(\delta PV_{exp}\right)}^{2}}$$

where *δ*PV_unexp_ and *δ*PV_exp_ are the uncertainties related to the pixel values for the calibration films before and after the irradiation, respectively. For both methods, this uncertainty is composed of two terms:

1. *σ*_A_, the experimental uncertainty (type‐A error) associated with the scanning measurement (uniformity and reproducibility in scanner acquisition, warming up effects); and
2. *σ*_B_, the statistical error due to the fact that the pixel values obtained in the calibration step are averaged on 1 × 1 cm^2^ ROIs (˜160 points).

These two error types were statistically added to produce the pixel value indetermination:

(11) $$\delta PV=\sqrt{\sigma_{A}^{2}+\sigma_{B}^{2}}$$

By scanning the films in accordance with the recommendations reported in paragraph A, it was possible to evaluate *σ*_A_ equal to 1% of the measured pixel value,^10^, ^12^ while *σ*_B_ was obtained by calculating the standard deviation on the 1 × 1 cm^2^ ROI.

For the SS method, *σ*(x_i_) was obtained by applying the error propagation law to Equation (2):

(12) $$\sigma_{SS}\left(x_{i}\right)=\sqrt{\sigma_{RC}{\left(x_{i}\right)}^{2}+\sigma_{GC}{\left(x_{i}\right)}^{2}+2\sigma_{RC,GC}\left(x_{i}\right)}$$

The covariance term *σ*_RC,GC_(x_i_), evaluated according to Equation (14), is present due to the fact that red and green channel values are derived from the same scan, so they have to be considered as correlated quantities.^20^, ^21^

(13) $$\sigma_{RC,GC}\left(x_{i}\right)=\sum_{i=1}^{k}\frac{\left(x_{i}^{RC}-{\overset{¯}{x}}^{RC}\right)\left(x_{i}^{GC}-{\overset{¯}{x}}^{GC}\right)}{k}$$

where ${\overset{¯}{x}}^{RC}$ and ${\overset{¯}{x}}^{GC}$ represent the average net optical density values obtained for the red and green channel, and k is the number of film pieces exposed to the same dose during the calibration step.

The size of the uncertainties related to the interfilm uniformity (Δ_film_, point 2) was determined by calculating the dispersion of the netOD values produced, by exposing five film pieces belonging to different sheets of the same batch to a 6 MV photon beam and delivering a dose of 2 Gy. Intrinsic dishomogeneities in the pixel values distribution (intrafilm uniformity) were corrected in the analysis of irradiated films by subtracting the unirradiated film from each irradiated film.

Finally, the fit uncertainty *σ*_fit_ was determined by the mean percentage error method, namely by computing the average of percentage errors by which our fitting model y_i,fit_ differs from actual values y_i,sper_:

(14) $$\Delta_{fit}=\frac{100\%}{n}\sum_{i=1}^{n}\frac{|y_{i,fit}-y_{i,sper}|}{y_{i,sper}}$$

where *n* is the total number of calibration dose points.

### 2.E Film QA verification and gamma analysis

The new SS method and the conventional SC method were compared by implementing the selected fit function in a QA protocol verifying patient‐specific dose distributions delivered with a Cyberknife system (version 9.6, Accuray, Sunnyvale, CA, USA). Clinically administered dose distributions were transferred to an Easy Cube phantom (Sun Nuclear, Melbourne, FL, USA), maintaining both the treatment beams ballistic and the number of Monitor Units (MU).

A Gafchromic EBT3 film was fixed in the phantom between the two central slabs, orthogonal to the craniocaudal axis of the treatment couch, and with its center in correspondence to the center of the slabs.

Four reference points corresponding to the film corners were marked on the slab surface to ensure accurate film position reproducibility. The CT scan of the phantom was then acquired. Film positioning corresponding to the planning target volume (PTV) was obtained by automatically aligning the mass centers of the two structures (Cyberknife Multiplan Treatment Planning System, version 4.6). The correct phantom setup on the treatment couch was ensured by matching, with sub‐millimeter geometric precision, the position of 8 fiducials (located in two different slabs, cranial and caudal, in relation to the fixed film position) on the digitally reconstructed radiographs and on the live images.

The planar dose distributions calculated by the treatment planning system (TPS) and the two film images (before and after irradiation) were imported into a Matlab homemade software (Math Works, Natick, MA, USA) structured to execute the following steps:

1. apply a median filter to the unirradiated image;
2. calculate the film net optical density and the film SS value;
3. apply the fit function to produce the film dose distribution for SC and SS;
4. coregister the film and the TPS dose distributions adjusting for possible rotational/translational displacements. The matching process is based on the spatial correlation method and is able to correct displacement variations with a precision of 0.5° and 0.25 mm;
5. evaluate the agreement between the two dose distributions in terms of gamma analysis^22^ for both the SC and the SS approaches. The comparison was carried out with ΔD_M_ = 3% −Δd_M_ = 1 mm and ΔD_M_ = 4%−Δd_M_ = 1 mm as acceptance criteria.

Before being used for patient QA verifications, the protocol was validated by verifying agreement of the two simple plans composed by a single beam (collimator aperture 6 cm) and prescription doses of 5 and 15 Gy.

Statistical significance of the gamma value differences between the SC and SS methods, determined using the Wilcoxon signed‐rank test for paired samples, was assessed. Differences were considered significant for *P* < 0.05.

Moreover, to further investigate the differences between SC and SS film dosimetries, the 20c Gy/1 mm gamma analysis was also performed by fixing a dose threshold equal to 4 Gy, and comparing SC and SS gamma distribution values for doses below and above this threshold.

Furthermore, as far as doses higher than 4 Gy were concerned (namely doses inside the PTV), an absolute dose measurements was performed using a 0.01 cm^3^ volume ionization chamber (CC01/TNC SN‐8911, IBA‐Dosimetry, Schwarzenbruck, Germany), designed for measurements of small fields with high dose gradients such as stereotactic fields, as a reference dosimeter. The CC01 chamber was positioned in a dedicated slab in the center of the Easy Cube phantom and aligned to the mass center of the PTV, corresponding to the center of the Gafchromic film, according to the same automatic procedure mentioned at the beginning of this paragraph. In this way, the same point dose in the patient's dose distribution was measured using both ionization chamber and Gafchromic film. This verification was performed only for those patients of the cohort presenting differences in the dose values estimated by SS and SC in the center of the Gafchromic film. Other localizations could not be taken into consideration because of the automatic alignment of the CC01 chamber limited to the center of the PTV.

## 3 Results

### 3.A Calibration curves

**Figure 1:**
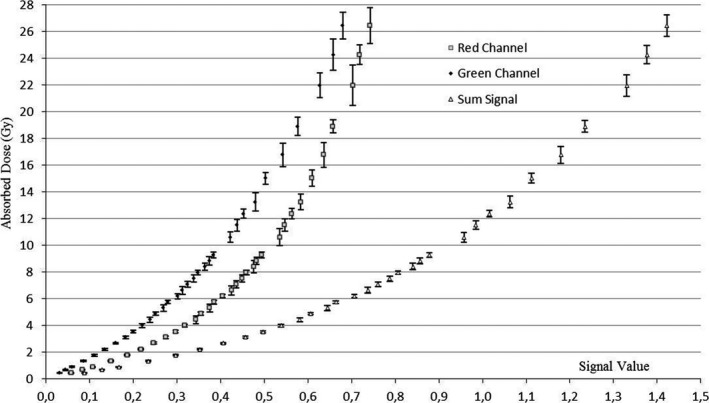
Calibration curves for red channel, green channel, and sum signal obtained by analyzing the batch #AO40411301

The calibration curves obtained simultaneously irradiating the two films belonging to the batch #AO40411301 are shown in Fig. 1 for RC, GC and SS.

The error bars associated with the dose values were calculated using Eq.(5).

**Figure 2:**
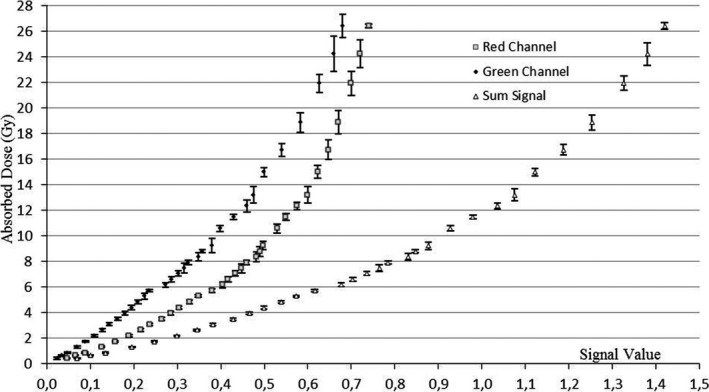
Calibration curves for red channel, green channel, and sum signal obtained by analyzing the validation batch

In Fig. 2, the reproducibility of the curve trends displayed in Fig. 1 was confirmed by the analysis of films belonging to the validation batch (#AO4041203).

### 3.B Dose resolution analysis

**Figure 3:**
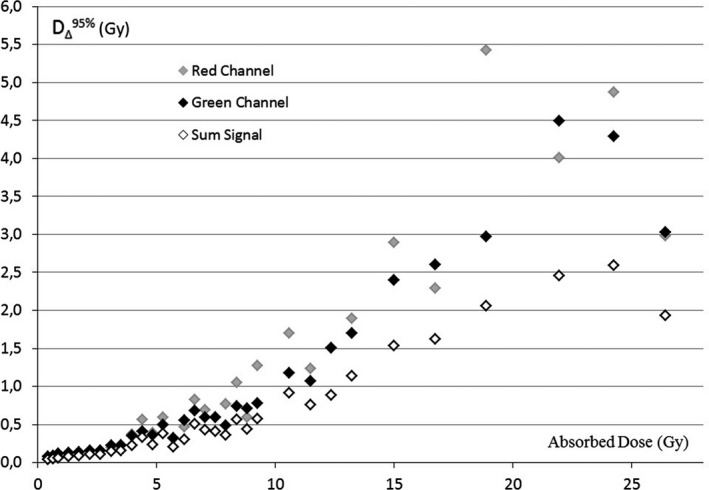
Dose resolution for red channel, green channel, and sum signal obtained analyzing the batch #AO40411301

In Fig. 3, the dose resolution values obtained by Eq. (4) for a level of confidence of 95% (D_∆_^95%^) are reported as a function of absorbed dose (Gy) for RC, GC, and SS.

**Figure 4:**
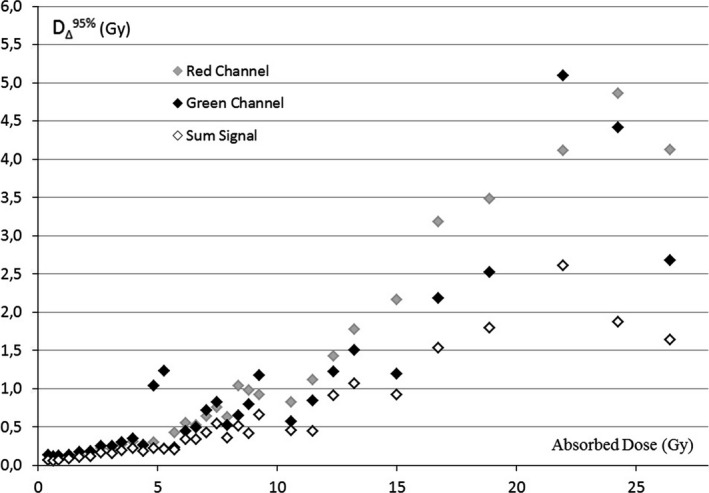
Dose resolution for red channel, green channel, and sum signal obtained analyzing the validation batch

A comparable trend has been detected by conducting the analysis on the validation batch, as shown in Fig. 4.

When looking at doses typical for most of the conformal and intensity modulated treatments (~2–3 Gy), the SS, RC, and GC methods are characterized by similar dose resolution. In comparison with the two single channels, the ability of SS to detect levels of dose differences improves for higher absorbed doses (> 4 Gy), with D_∆_^95%^ values below 50 cGy, up to 9 Gy, and remaining below 1 Gy for doses up to 13 Gy.

The impact of the improved resolution for the SS method in the framework of a patient‐specific QA protocol was assessed in this study by comparing gamma values as reported in section E.

### 3.C Dose calibration

Table 1 reports the AICc values calculated for the functional forms discussed in this section. The comparison analysis among the various functions *f(x)* was carried out separately for RC, GC, and SS on the batch #AO40411301.

**Table 1:** AICc values for RC,GC, and SS, where K is the number of parameters used in the function

Functional form	K	AICc (RC)	AICc(GC)	AICc (SS)
*f(x) = c*_1_ exp(*c*_2_ *x*) + *c*_3_ exp(*c*_4_ *x*)	4	10.78	10.13	9.87
*f(x) = c*_1_ *x* ^3^ + *c*_2_ *x* ^2^ + *c*_3_ *x* + *c*_4_	4	14.36	10.36	10.02
*f(x) = c*_1_ *x* ^4^ + *c*_2_ *x* ^3^ + *c*_3_ *x* ^2^ + *c*_4_ *x* + *c*_5_	5	13.75	12.64	12.45
*f(x) = c*_1_ *x* ^*5*^ * + c*_2_ *x* ^*4*^ + *c*_3_ *x* ^3^ + *c*_4_ *x* ^2^ + *c*_5_ *x* + *c*_6_	6	15.50	15.36	15.08
*f*(*x*) = *c*_1_ *x + c*_2_ */c*_3_ *x* ^2^ + *c*_4_ *x* + *c*_5_	5	16.23	21.25	12.54

Although some functional forms are characterized by similar results, it is shown by the table that the double exponential presents the lowest AICc values for all three methods under investigation. The same conclusion was drawn also from the AICc values deriving from the validation batch. The double exponential function was consequently used to fit the calibration data into our high‐dose verification protocol.

### 3.D Accuracy estimation of the dosimetric protocol

Table 2 summarizes the uncertainty values for the three main sources previously analyzed and gives the overall accuracy Δ_tot_ of a dosimetric protocol implementing each of the three methods under investigation.

**Table 2:** Uncertainty values and related overall accuracy for SC and SS methods

Type error	Red channel	Green channel	Sum signal
Δ_meas_	1.32%	1.87%	1.30%
Δ_film_	1.10%	1.13%	0.92%
Δ_fit_	0.99%	1.11%	0.82%
Δ_tot_	1.98%	2.45%	1.79%

Values shown in the table for Δ_meas_ [Eq. (10)] are obtained as an average of the percentage errors computed for all of the calibration dose films.^23^, ^24^

The addition of the covariance term [see eq. (14)] to the Δ_meas_ calculation results in the increment of Δ_meas_ value from 1.12% to 1.30%.

### 3.E Film QA verification and gamma analysis

In Table 3, the percentage of *γ* points< 1 obtained by irradiating a single beam at prescription doses of 5 and 15 Gy is reported.

**Table 3:** Gamma values for single beam

Single beam	*γ*<1 (3%/1 mm)		*γ*<1 (4%/1 mm)	
	SC	SS	SC	SS
5 Gy	93.11%	95.21%	99.87%	99.95%
15 Gy	99.10%	99.25%	99.76%	99.77%

The results of the validation test show that a high percentage of points satisfy the pass rate *γ* < 1 for both SC and SS. The gamma values > 1, analyzed using the gamma angle tool^25^ implemented in the homemade Matlab software, are due to the dose difference component of the gamma test.^22^

The comparison between the SC and SS methods in terms of gamma analysis for patient QA verification was performed for 20 intracranial lesions, with maximum planned doses per fraction ranging from 5 to 21 Gy. Average dimensions of the radiosurgical target in the axial plane were 32 mm (range: 15–55 mm) and 25 mm (range 11–43 mm) for anterior–posterior and left–right directions, respectively. Table 4 shows the mean percentage of *γ* values < 1, between 1 and 1.5, and > 1.5 obtained for the 20 lesions analyzed in this study.

**Table 4:** Gamma values for 20 patient plans

Method	*γ* [3%/1 mm]			*γ* [4%/1 mm]		
	γ < l	1 < γ < 1.5	γ > 1.5	γ < l	1 < γ < 1.5	γ > 1.5
Sum signal	95.03%	4.17%	0.80%	97.24%	2.36%	0.39%
Single channel	88.41%	9.43%	2.15%	93.58%	5.89%	0.52%

The percentage of dose distribution points with *γ* < 1 is higher for the SS method compared to the SC method: 95.03% vs. 88.41% for 3%/1 mm acceptance criteria, 97.24% vs 93.58% for 4%/1 mm acceptance criteria, respectively. The statistical significance of these differences is confirmed by a *P* value equal to 0.014 for 3%/1 mm and 0.049 for 4%/1 mm acceptance criteria.

Table 5 reports the results obtained for the 20 cGy/1mm gamma analysis limiting film doses from 0 to a threshold value of 4 Gy and considering doses greater than 4 Gy.

**Table 5:** Gamma values for 20 plans limiting film doses to a maximum value of 4 Gy

Method	γ < l [20 cGy 1 mm; D < 4 Gy]			γ <l [20 cGy 1 mm; D > 4 Gy]		
	Average	Standard deviation	*P* value	Average	Standard deviation	*P* value
Sum signal	92.77	2.79	0.424	91.15	6.36	0.027
Single channel	91.37	4.89		85.33	9.80	

The difference between the two methods is statistically significant only for doses greater than 4 Gy. Table 6 shows the results of the dose measurements performed with the CC01 ionization chamber in the center of the PTV for eight selected patients. The estimation of the dose values reported for SS and SC was obtained as an average of the values of the area corresponding to the planar dimension of the CC01 ion chamber. The last two columns give the percentage difference between the chamber reading and the corresponding dose value estimated using the irradiated Gafchromic film for SS and SC, respectively.

**Table 6:** Comparison between ion chamber reading (CC01) and SS and SC dose value estimation in the center of the PTV. The mean dose values calculated by the TPS in the whole chamber volume are also reported for further information

Patient	CCOl(Gy)	SS (Gy)	SC (Gy)	TPS (Gy)	SS‐CC01/CC01 (%)	SC‐CC01/CC01 (%)
1	4.54	4.45	4.63	4.37	−1.98	1.98
2	4.29	4.43	4.65	4.74	3.26	8.39
3	6.47	6.63	7.00	6.44	2.47	8.19
4	4.70	4.96	5.26	4.70	5.53	11.91
5	4.86	4.83	5.07	4.86	−0.62	4.32
6	13.09	13.08	13.11	12.86	−0.08	0.15
7	12.99	13.01	13.23	12.61	0.15	1.85
8	15.37	14.36	13.64	14.16	−6.57	−11.26

This percentage difference is generally smaller for the SS method, with the exception of patient 1 for which SS and SC behaved similarly.

## 4 Discussion

### 4.A Dose–response curve

A preliminary visual examination of the data shown in Figs. 1 and 2 leads to the conclusion that larger signal spacing for consecutive dose values can be appreciated for the SS approach.

When considering doses greater than 4 Gy, RC and GC show signal values most frequently associated with overlapping error bars. Film sensitivity at high doses using the SC approach therefore seems very limited.

In general, signal values for the SS curve are widely spaced, producing a steeper trend and suggesting improved film sensitivity at higher doses with respect to the SC method.

Also, reproducibility was observed in the behavior of films belonging to different batches.

The spillover effect of this increase in sensitivity on the minimal separation of two contiguous doses, at which their most probable values are different within a given level of confidence, is quantified by the dose resolution analysis in the following paragraph.

### 4.B Dose resolution analysis

The resolution values illustrated in Figs. 3 and 4 confirm the published data reporting about the red and green channel responses to radiation exposure. In particular, the GC resolution was confirmed to be higher than the RC resolution for doses above 8–10 Gy.^1^, ^4^

Provided that dose resolution worsening is taken into consideration, the use of the SS method can be extended to higher dose levels, until saturation effects are observed. These effects were found by Borca et al. to occur from a dose level which varied for each specific channel (38 Gy for RC and above 40 Gy for GC).^4^

### 4.C Curve fitting

The AIC was chosen in particular as an alternative to the sum of residuals because it also takes into account the effective uncertainties *σ*_eff_(y_i_) and the number of parameters used in the fit function.

It demonstrates that, provided the most appropriate functional form is chosen, four parameters are sufficient to fit the whole film dose–response object of this study. It is therefore possible to adopt the SS method using only four calibration points, as advocated by the film vendor.^5^

### 4.D Accuracy estimation of the dosimetric protocol

The results obtained in terms of overall accuracy for individual channels (1.98% for RC and 2.45% for GC) are in agreement with the results obtained for the RC method by Ferreira (1.8%) and Martisikova (1.6%) who apply the law of error propagation, and by Huet et al. through Monte Carlo simulations (< 2%).^17^ As shown in Table 2, Δ_tot_ for SS is comparable to value obtained for RC and GC. Based on these results, the proposed high‐dose protocol implementing SS ensures a high degree of accuracy, comparable to the one achievable by the SC method.

### 4.E Film QA verification and gamma analysis

Dose tolerance levels ΔD_M_ used for gamma analysis were calculated as a percentage of the plan prescription dose, and they were chosen in consideration of the geometrical complexity of delivery (more than a hundred beams for each treatment plan) of the dose inhomogeneity within the target and of the estimated overall accuracy of the dosimetric protocol.^26^ The distance tolerance level Δd_M_ was chosen as 1 mm, taking into account the steep dose gradients over short distances that characterize radiosurgical treatments and the observations of accuracy of both the experimental film setup and the fiducial tracking used for dose delivery reported in the section E of the Methods paragraph.

In general, the results contained in Table 3 for the gamma analysis of single beam are an indication of an extremely good agreement between measured and calculated dose distribution for SC and SS in the simple case of a nonmodulated dose distribution, confirming the validity of the proposed protocol at low‐medium and high doses.

When modulated dose distributions are taken into consideration, as in the case of the 3%/1 mm and 4%/1 mm gamma results obtained for the 20 patients listed in Table 4, the SS method developed in this study shows higher effectiveness compared to the SC method.

This behavior is confirmed also if we apply the experimental uncertainties reported in Table 2 to the measured dose distributions (± 1.98% for the RC, ± 2.45% for the GC, ± 1.79% for the SS), in fact the percentage of *γ* points < 1 decreases by the same quantity (equal to ~5% in the 3%/1 mm case and equal to ~3% in the 4%/1 mm case) for sum signal and single channel.

Furthermore, the analysis of the 20 cGy/1 mm gamma results contained in Table 5 leads to the conclusion that SS and SC can be considered equivalent at doses lower than 4 Gy, while the SS performs better for doses higher than 4 Gy. The superiority of SS at high doses is confirmed also by the ion chamber measurements in the center of the PTV. In fact, from the data contained in Table 6 it can be appreciated that the percentage difference with respect to the estimated dose values is generally smaller for the SS method.

## 5 Conclusions

In recent years, several protocols have been developed to improve SC film dosimetry accuracy.^2^, ^17^ Some focus on the digitization procedures^16^, ^24^ while others deal with the assessment of the different uncertainty sources affecting the measurements.^23^, ^27^, ^28^ However, the SC reduced sensitivity still limits the accuracy of the resulting dosimetric analyses for high‐dose treatment plans.

A different approach to high‐dose verifications is to apply a scaling factor to the delivered dose, and reduce it to a value that falls in the film's sensitive range. The problem with this approach lies in the fact that for dosimetry of complex delivery techniques it does not assure the administration of the minimum number of MU needed for the accelerator to achieve a stable output during treatment irradiation.^29^

The results of this study confirm the suitability of the SS method applied to EBT3 films for the dosimetry of state‐of‐the‐art precision RS/HRT treatments where multiple beams, delivery angles and LINAC movement are used for optimal dose conformation to the target, and overcoming the limitations of dose scaling or color channel switching procedures.

The concept of dose resolution, used to compare the effectiveness of the SS to the SC approach, also gives valuable information about the accuracy of dose distribution verifications as a function of delivered dose in the clinical evaluation of patient dose distribution QA.

The SS method can thus be considered an effective and promising method to improve dosimetric accuracy in the framework of the RS‐HRT patient‐specific QA protocol.
